# Enzymatic Degradation of PLA: Preferential Degradation of the Amorphous Fraction

**DOI:** 10.3390/polym17223042

**Published:** 2025-11-17

**Authors:** Sarita Shrestha, Michael Snowdon, David B. Levin

**Affiliations:** 1 Department of Biosystem Engineering, University of Manitoba, Fort Garry Campus, 75 Chancellors Circle, Winnipeg, MB R3T 5V6, Canada; sarita.shrestha@umanitoba.ca (S.S.); david.levin@umanitoba.ca (D.B.L.); 2 Sustainable Packaging Team, Automotive and Surface Transportation, National Research Council Canada, Advanced Manufacturing Research Facility, 2690 Red Fife Road, Centreport, Winnipeg, MB R4B 0A6, Canada; michael.snowdon@nrc-cnrc.gc.ca

**Keywords:** Polylactic acid (PLA), enzymatic degradation, Proteinase K, polymer structure

## Abstract

Polylactic acid (PLA), a widely used biobased biopolymer, is highly resistant to biodegradation under ambient conditions, contributing to persistent plastic pollution and posing potential environmental and health risks. This study investigates the enzymatic degradation of PLA by Proteinase K, a proteolytic hydrolase enzyme with the ability to degrade PLA, and explores the underlying mechanisms for degradation. Both amorphous and semi-crystalline PLA were treated with Proteinase K (2 mg/mL) at 37 °C over varying time periods. PLA degradation was evaluated using multiple techniques, including weight loss measurement, pH reduction, quantification of lactic acid monomer release by High-Performance Liquid Chromatography (HPLC), surface morphology analysis through Scanning Electron Microscopy (SEM), changes in thermal properties by Differential Scanning Calorimetry (DSC), and structural changes by X-Ray Diffraction (XRD). The data revealed that the degradation of amorphous regions of the PLA polymer was faster and more extensive than the crystalline regions of the polymer. Repeated enzymatic treatments significantly enhanced the degradation rate. Furthermore, Proteinase K showed a clear preference for degrading amorphous regions of the PLA, as evidenced by higher weight loss, sharper pH decline, higher lactic acid production, and more pronounced surface disruptions, such as visible gaps between degraded oligomer structures.

## 1. Introduction

Polylactic acid (PLA), an aliphatic polyester, is one of the most widely used and available biopolymers. PLA is considered a ‘biopolymer’ because it is derived from renewable, agricultural resources such as corn or other carbohydrate sources [1,2,3], even though it is chemically synthesized [4]. PLA has high mechanical strength, is biocompatible, industrially compostable, non-toxic, non-carcinogenic, and easy to fabricate [5,6]. The physical, mechanical, and thermal properties of PLA make it suitable for a wide range of commercial products, increasing the demand and production of PLA significantly over the past decade [1,6]. Based on the Bioplastic Market Development Update 2023, PLA production was 31.0% among all the biodegradable bioplastics and is estimated to increase to 43.6% by 2028 [7]. This will subsequently increase the amount of plastic waste as it is not readily recycled and leads to leakages in both terrestrial and aquatic environments when mismanaged, polluting the surroundings.

The PLA degradation presents challenges; it degrades easily in industrial composting systems at higher temperatures (58 ± 5 °C) but is highly resistant to biodegradation under mesophilic temperatures [3,8]. The degradation of PLA polymers also depends on various factors, such as crystallinity, enantiomeric composition, size, molecular weight of the polymers, and other environmental conditions (pH, temperature, presence of microorganisms, types of microorganisms, etc.) [1,3,8,9,10]. The high demand for single-use compostable plastic, the inability to degrade at mesophilic temperatures, and the worldwide issues with plastic waste management have led PLA to contribute to global plastic pollution [5]. Therefore, there is a need to find a way to minimize plastic pollution, including that of PLA.

A number of studies have reported that PLA can be degraded by hydrolytic enzymes such as protease, esterase, lipase, and chitinase, which are produced by microorganisms [11,12,13,14]. The biodegradation of PLA polymer by using enzymes is more efficient and beneficial than the chemical or physical methods, as enzymatic degradation is a mild process that does not generate harsh chemicals and uses less energy [11,12]. Lipase and Proteinase K were used for the degradation of poly(L-lactide) and poly(3-hydroxybutyrate-co-4-hydroxybutyrate) polymer blends prepared by melt compounding [15], and both Esterase ABO2449 and Esterase RPA1511 efficiently hydrolyzed PLA and were specific to D, L-PLA, but not L-PLA or D-PLA [16]. Another study demonstrated that the enantiomeric composition of PLA affects degradation. The enzymatic degradation of PLA with 40/60 L-lactide/D-lactide PLA was more prominent than 25/75 and 10/90 L-lactide/D-lactide PLA [9]. Hydrolytic degradation of PLA occurs mainly due to two mechanisms, heterogeneous and homogeneous erosion, which initiate in amorphous regions, cleaving the ester bond [1].

In this study, we exploited a commercial enzyme, Proteinase K, which is a fungal serine protease produced by *Tritirachium album*. *T. album* was previously shown to cleave PLA polymer chains and accelerate the degradation of L-PLA [17]. Protease K belongs to the Subtilisin enzyme family and has a typical serine protease catalytic triad structure (Asp-His-Ser), with a wide range of substrate specificity [18]. It preferentially hydrolyzes the ester and peptide bonds adjacent to the C-terminal of polypeptide chains, as well as sulfur-containing, hydrophobic, and aromatic amino acids [11,19,20]. Proteinase K can remain active in high-temperature environments and a wide range of pH conditions [21].

The mechanism of PLA degradation by Proteinase K remains unclear [11]. The goal of this study was to explore the degradation mechanisms and to determine and compare amorphous or semi-crystalline PLA polymer degradation using Proteinase K at 37 °C and pH 8.5. PLA degradation was evaluated using multiple techniques, including weight loss measurement, pH reduction, quantification of lactic acid monomer release by High-Performance Liquid Chromatography (HPLC), surface morphology analysis through Scanning Electron Microscopy (SEM), changes in thermal property by Differential Scanning Calorimetry (DSC), and structural changes by X-Ray Diffraction (XRD).

## 2. Materials and Methods

### 2.1. Sample (PLA Film) Preparation

PLA cast films (220 µm thick) with a low percentage of crystallinity used in this study were prepared and provided by the National Research Council Canada (NRC). The low crystallinity PLA films had ~0 to 3% crystallinity measured via DSC and are referred to as “amorphous PLA”. Ingeo PLA 2500HP (Nature Works, Minnesota, USA) was used as a semi-crystalline PLA, which contains greater than 99% L-isomer and 0.25–0.5% D-isomer of the PLA. An annealed form of PLA (semi-crystalline PLA) films had ~47 to 50% crystallinity based on DSC analysis, which are referred to as “semi-crystalline PLA”. The semi-crystalline PLA films were also provided by NRC and were prepared by annealing the PLA film in a hot air oven at 90 °C for 1 h. Approximately 1.5 cm × 1.25 cm PLA films were cut and cleaned with a clean wipe (Kimwipe), followed by cleaning with methanol and deionized water. Then, again, the PLA film pieces were wiped with a clean wipe and dried in desiccators for 48 h.

### 2.2. Weight Loss

After drying, each PLA film piece was weighed on an analytical balance (Mettler Toledo, Switzerland, B923777225:ME104). The initial weight of the PLA piece was denoted as Wi. The PLA samples were placed into the glass tubes with lids containing enzyme solutions (2 mg/mL in Tris-HCl buffer of pH 8.5). The tubes were then incubated at 37 °C for specific periods of time: 1 h, 2 h, 4 h, 8 h, 1 day, 2 days, 3 days, 4 days, and 8 days. In a separate experiment, PLA samples in glass tubes were incubated at 37 °C for 4 days (96 h). The enzyme solution was changed every four days, and incubation was repeated 3 times, for a total incubation period of 16 days. The PLA film pieces were taken out of the enzyme solution after specific times, washed with ultrapure water 5–7 times, and then dried in the desiccator at room temperature for 48 h. Once dried, they were again weighed with an analytical balance. The weight of the PLA after treatment was noted as Wf. The weight loss (D) of the PLA films was calculated as follows: D (%) = (Wi − Wf) × 100/Wi, where Wi and Wf represent the initial and final weights of the PLA film, respectively [22].

### 2.3. pH Change

The main product resulting from PLA hydrolysis is lactic acid, which leads to changes in pH. Therefore, the pH value of the Tris-HCl buffer was measured at the beginning and end of the experiment with a digital VWR sympHony SB80PI benchtop pH meter [22].

### 2.4. Lactic Acid Production

After treating PLA film with Proteinase K for specific periods of time, the PLA films were removed, and the concentration of lactic acid in the reaction supernatant solution was quantified by HPLC: Water model 1515 with a Refractive Index detector (model 2414), an Aminex HPX-87H column (catalog 125-0140), and a micro-guard column H (catalog 125-0129). The mobile phase was 5 mM H_2_SO_4_, the detector temperature was 45 °C, and the flow rate was 0.60 mL/min. The supernatants were passed through 0.22 μm microporous filters to remove insoluble impurities before performing HPLC analysis. Prior to this, different concentrations of sodium lactate were analyzed using HPLC for a lactic acid standard graph.

### 2.5. Surface Morphology of PLA Film

The change in surface morphology was observed using an FEI Quanta 650 FEG Environmental Scanning Electron Microscope (SEM). The PLA film samples were mounted on SEM stubs with double-sided carbon tape. The samples were coated with a thin layer (20 nm) of gold and palladium alloy (60:40 percent) in a Denton Sputter Coater for 90 s. SEM images were obtained with an accelerating voltage of 5 kV.

### 2.6. Thermal Properties of the PLA Film

The thermal properties of the PLA films were analyzed by Differential Scanning Calorimetry (DSC) using a Discovery DSC 250, TA Instruments, Waters LLC, New Castle, DE, USA. Approximately 10–15 mg of PLA film was sealed in pans with lids and subjected to a heat–cool–heat cycle at 10 °C/min. The degree of crystallinity of the samples was determined by the following formula [22]: Xc (%) = (∆Hm − ∆Hc) × 100/∆H^0^m, where Xc is the degree of crystallinity, ∆Hm is the enthalpy during melting, ∆Hc is the exothermic peak enthalpy, and ∆H^0^m is the enthalpy for the normalized enthalpy values (J/g) for 100% crystalline PLA polymer, which was reported to be 93.7 J/g in the literature [23].

### 2.7. Structural Changes in the PLA Films

The structural changes in PLA samples were further analyzed by X-Ray Diffraction (XRD) analysis using a D5000 diffractometer (Bruker, formerly Siemens) equipped with a Cu X-ray tube and graphite monochromator (to remove K-Beta). The diffractogram was recorded at room temperature and was scanned from 5 to 50° at 2-theta using a 2°/min scan rate with reporting every 0.05°. The X-ray beam was directed parallel to the film surface. The Crystallinity Index (Xc%) was calculated from the ratio of the area under the crystalline peak to the total area of crystalline and amorphous peaks, using the following formula: Crystallinity Index (Xc%) = Ac × 100/(Ac + Aa), where Ac is an area under the crystalline peaks (sharp/narrow) and Aa is the area under the amorphous halo (broad hump).

### 2.8. Statistical Analysis

All the experiments were conducted in triplicate, and the data were inserted into an Excel file and analyzed. Statistical significance was analyzed using one-way analysis of variance (ANOVA) followed by Tukey’s post hoc test. The *p*-value was set at <0.05 as the significance level threshold.

## 3. Results

### 3.1. Weight Loss

**Figure 1:**
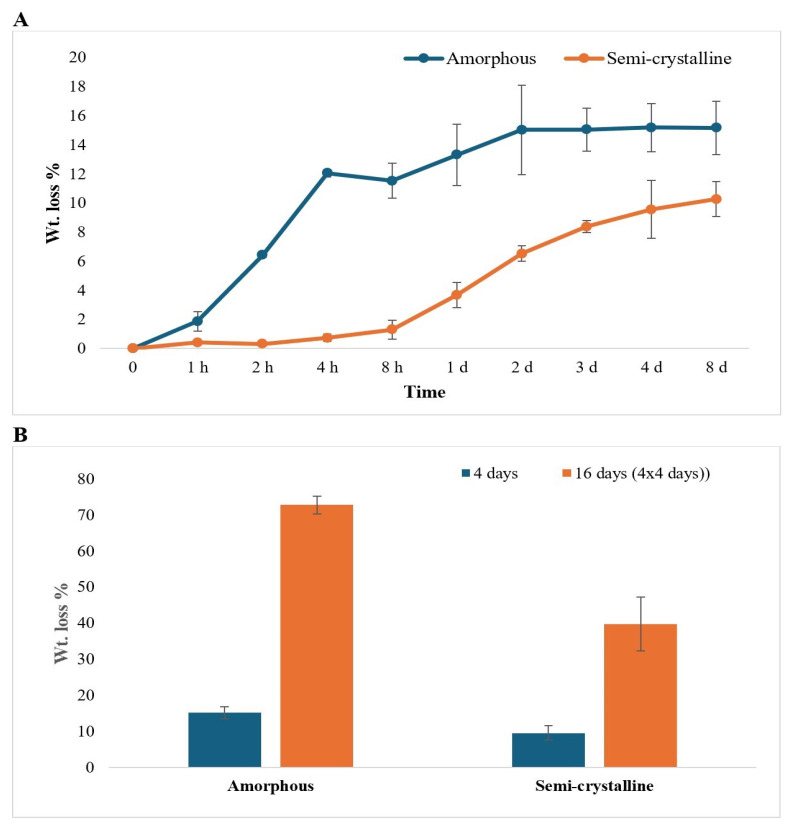
Weight loss percentage of (**A**) amorphous and semi-crystalline PLA for 0 h to 7 d (h: hour; d: day) and (**B**) amorphous and semi-crystalline PLA for 4 days and 16 days (4 × 4 days). Enzyme treatment for 16 days represents the experiment that was repeated 4 times, with 4 days of incubation between each treatment. The data are represented as means of three (triplicate) independent experiments, with error bars representing the standard deviations.

The weight loss of the PLA films was found to increase with time for both amorphous and semi-crystalline PLA films. The weight loss for amorphous PLA film was observed to be greater (15.2% in 8 days of treatment) compared to semi-crystalline PLA film (10.3% in 8 days). The weight losses were not significantly different after 4 h of enzymatic treatment for amorphous PLA and 2 days of treatment for semi-crystalline PLA film (Figure 1A). After repeated addition and incubation of PLA with Proteinase K, weight loss of the amorphous PLA samples was more than 5 times (72.7%) greater compared to amorphous PLA samples exposed to a single enzyme treatment (15.2%), while weight loss of the semi-crystalline PLA samples was 4 times greater (39.7%) than semi-crystalline PLA samples exposed to a single enzyme treatment (9.6%%) (Figure 1B).

### 3.2. pH

**Figure 2:**
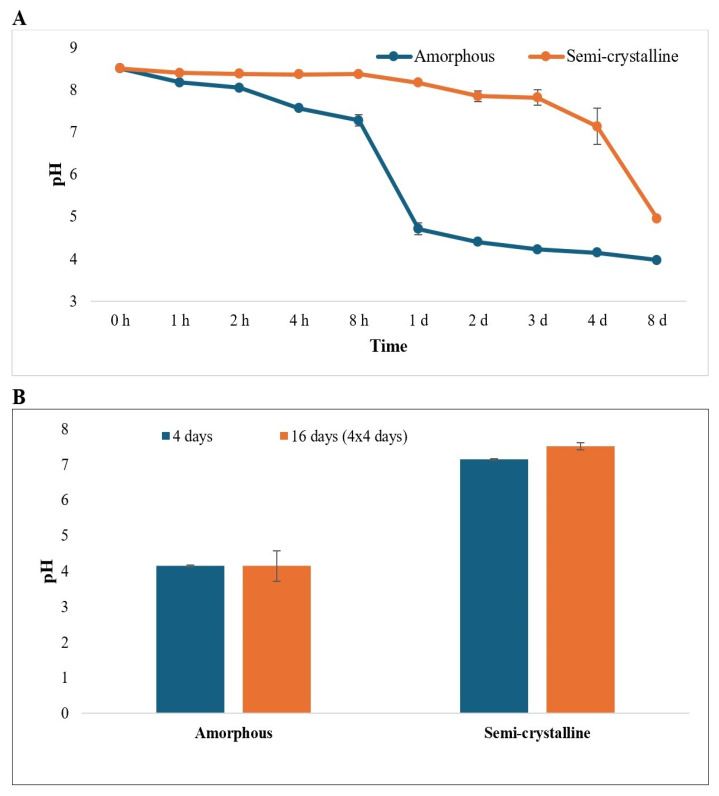
Changes in pH after enzymatic treatment of (**A**) amorphous and semi-crystalline PLA for 0 h to 7 d (h: hour; d: day) and (**B**) amorphous and semi-crystalline PLA for 4 days and 16 days (4 × 4 days). Enzyme treatment for 16 days represents the experiment that was repeated 4 times, with 4 days of incubation between each treatment. The data are represented as means of three (triplicate) independent experiments, with error bars representing the standard deviations.

The initial pH of the Tris-HCl buffer was 8.5, but it decreased over time following incubation of PLA samples with Proteinase K. The pH of the reactions in which amorphous PLA was incubated with Proteinase K decreased from 8.5 to 3.97, while the pH of reactions in which semi-crystalline PLA was incubated with Proteinase K decreased from 8.5 to 4.95 (Figure 2A). This decrease in pH was due to the production of lactic acid released during the enzymatic hydrolysis of the PLA polymer. The pH of the enzyme solution did not decrease significantly for either amorphous or semi-crystalline PLA after repeated addition and incubation of PLA samples with Proteinase K (Figure 2B).

### 3.3. Lactic Acid Production

**Figure 3:**
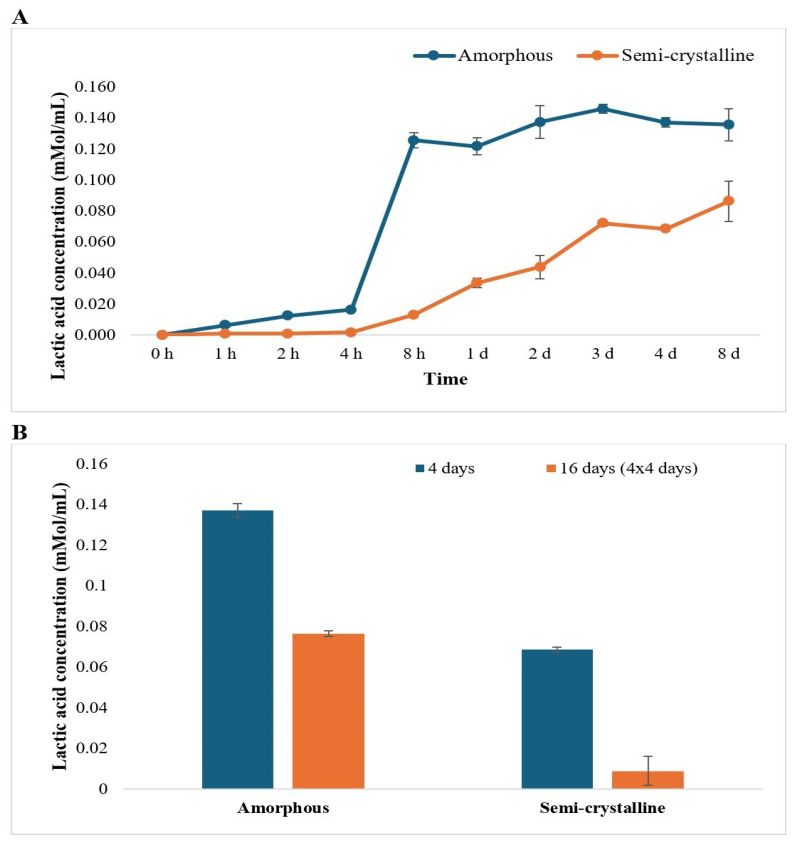
Lactic acid concentration detected during Proteinase K treatment of (**A**) amorphous and semi-crystalline PLA for 0 h to 8 days (h: hour; d: day) and (**B**) amorphous and semi-crystalline PLA for 4 days and 16 days (4 × 4 days). Enzyme treatment for 16 days represents the experiment that was repeated 4 times, with 4 days of incubation between each treatment. The data are represented as means of three (triplicate) independent experiments, with error bars representing the standard deviations.

The lactic acid concentrations were quantified using HPLC, based on a standard curve generated from known lactide concentrations. Standard samples of lactide on HPLC produced a characteristic peak at a retention time of around 12.9 min, referring to lactic acid. Similarly, the HPLC results of the solution obtained after the Proteinase K-mediated degradation of PLA films illustrated a peak at the same retention time, referring to a lactic acid monomer as the product of hydrolysis [1]. Lactic acid concentrations were observed to increase with time for both amorphous and semi-crystalline PLA. The concentrations of lactic acid (LA) generated by Proteinase K treatment were significantly different for amorphous versus semi-crystalline PLA. However, the LA concentrations for amorphous PLA were not significantly different after 8 h, whereas LA concentrations continued to increase after 72 h (3 days) for semi-crystalline PLA (Figure 3A).

After repeatedly incubating with the enzyme, LA concentrations were greater for amorphous PLA compared to semi-crystalline PLA. Interestingly, the LA concentrations in repeatedly treated samples were lower than those observed in samples exposed to a single round of Proteinase K treatment (Figure 3B).

**Figure 4:**
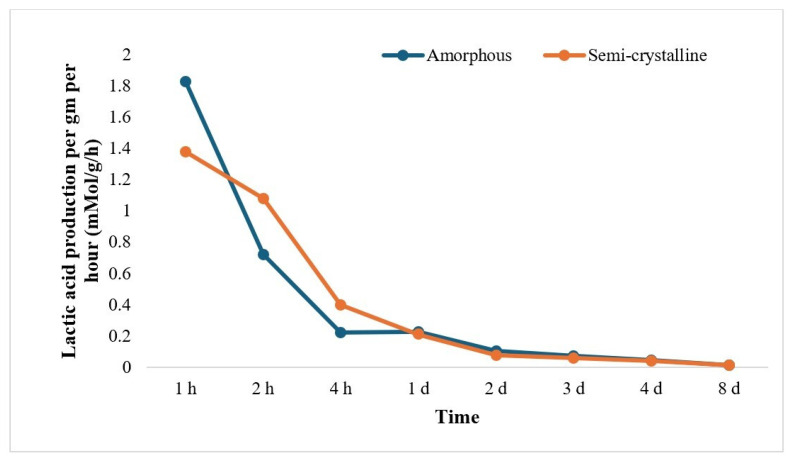
Lactic acid production per gram per hour from 0 h to 7 d (h: hour; d: day) of Proteinase K treatment. The data is represented as means of three (triplicate) independent experiments.

The rate of LA release (mMol/g/h) was calculated from the weight loss per gram of PLA film and LA concentration from HPLC data and was found to decrease as the incubation time increased. The trend of LA release per gram and per hour was observed to be at the maximum in the beginning (~1.8 mMol/g/h for amorphous PLA and ~1.4 mMol/g/h for semi-crystalline PLA) and was almost negligible after 2 days of treatment for both amorphous PLA and semi-crystalline PLA (Figure 4).

### 3.4. Thermal Properties of PLA Films

**Figure 5:**
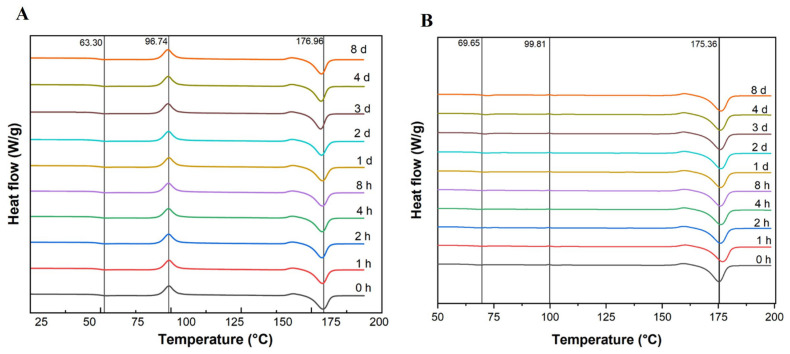
DSC thermograms of (**A**) amorphous and (**B**) semi-crystalline PLA films at different times (h, hour; d, day).

The DSC thermograms of both amorphous and semi-crystalline PLA films after treatment for different time periods showed negligible difference. The exothermic peak at 97 °C of amorphous PLA (Figure 5A) corresponding to the cold crystallization (also referred to as the temperature of cold crystallization) remained present over the treatment time, while a cold crystallization peak was not detected for semi-crystalline PLA (Figure 5B). An endothermic peak corresponding to the melting temperature, at ~175–177 °C, was observed for both amorphous and semi-crystalline PLA, with limited variability over the course of the treatment period.

**Figure 6:**
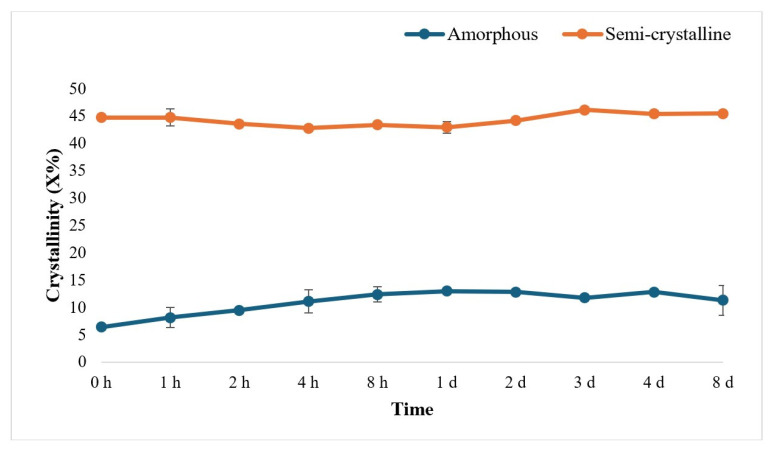
Percent crystallinity in amorphous and semi-crystalline PLA films after treatment with Proteinase K for different time periods (0 h to 8 d; h: hour; d: day), determined from the DSC data. The data are represented as means of three (triplicate) independent experiments, with error bars representing the standard deviations.

The trends for the crystallinity of PLA were analyzed from 0 h to 8 days of enzyme treatment of both amorphous and semi-crystalline PLA. The trend in crystallinity was observed to be not significantly different among treated semi-crystalline and amorphous PLA films (Figure 6).

### 3.5. Structural Changes in the PLA Films

**Figure 7:**
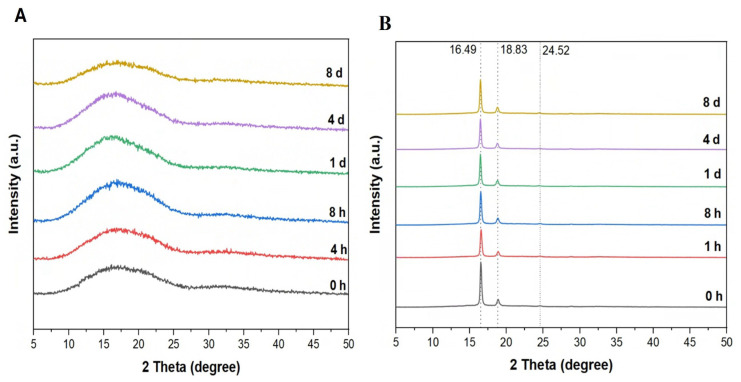
XRD spectra for (**A**) amorphous and (**B**) semi-crystalline PLA films at different times (h: hour; d: day).

The intensity of the diffraction peaks in XRD is related to the crystallinity. The diffractograms of the amorphous and semi-crystalline PLA films were very different, while the diffractograms of the amorphous films and the semi-crystalline films over 8 days of treatment were very consistent (Figure 7). The diffractogram of amorphous PLA films also illustrated the broad peak between ~9° and 27° and did not show any sharp peak (Figure 7A). In contrast, the diffractograms of semi-crystalline PLA films showed a sharp peak at ~16° and another smaller peak at ~18° (Figure 7B).

**Figure 8:**
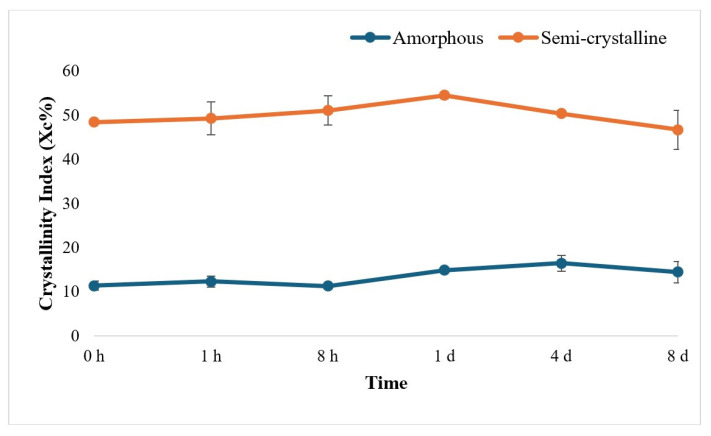
Crystallinity Index (Xc%) calculated from XRD data from 0 h to 8 d (h: hour; d: day). The data are represented as means of triplicate experiments with error bars of standard deviations.

Further, the Crystallinity Index of both amorphous and semi-crystalline PLA films was calculated from XRD data, and the trend in the Crystallinity Index was observed to be non-uniform and not significantly different (Figure 8).

### 3.6. Scanning Electron Microscopy (SEM)

**Figure 9:**
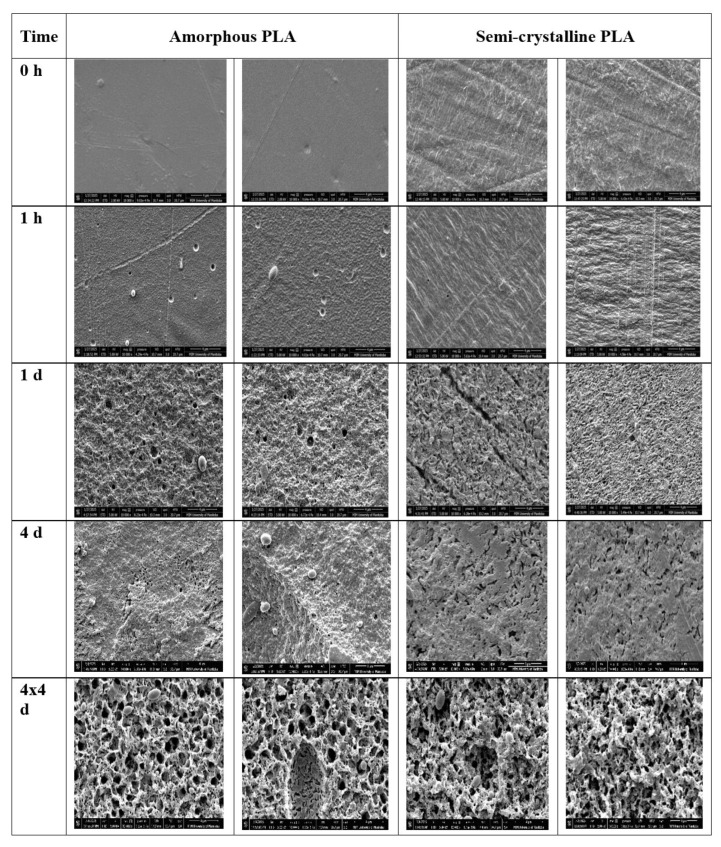
SEM micrographs (10,000× magnification) of amorphous PLA and semi-crystalline PLA after enzyme treatment for 0 h (i.e., without treatment), 1 h, 1 d, 4 d, and 4 × 4 d (h: hour; d: day), where 4 × 4 days denotes enzyme treatment repeated 4 times, with 4 days of incubation between each treatment.

SEM images revealed notable changes in the surface morphologies of both amorphous and semi-crystalline PLA films during Proteinase K treatment (Figure 9). The presence of irregular surfaces, cracks, and holes indicated increased surface roughness due to enzymatic degradation. The holes were observed earlier in amorphous PLA (1 h) compared to semi-crystalline PLA (4 h), while the cracks were mostly observed in semi-crystalline PLA. These surface features—roughness, cracks, and pores—were more prominent after repeated incubation in the enzyme solution, 3 times for 4 days (4 × 4 days). SEM images of Proteinase K-treated PLA at 0, 1, 4, 8, 24, 96, and 192 h, and after repeated Proteinase K treatment, are shown in Supplementary Figure S1.

**Figure 10:**
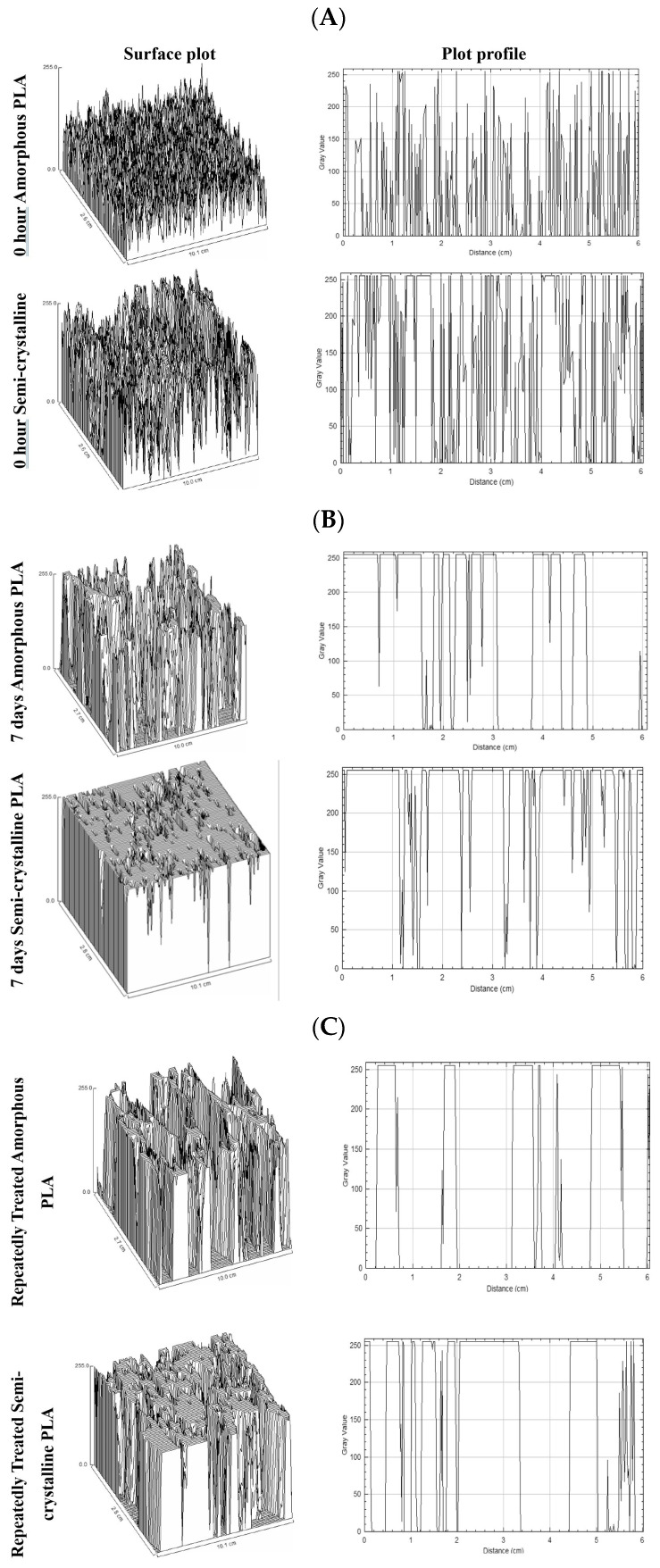
Visualization of the surface (surface plot) and cross-sectional (plot profile) structures of amorphous and semi-crystalline PLA films (**A**) before (0 h); (**B**) at 7 days; and (**C**) after repeated addition of Proteinase K.

Further analyses of SEM images using Image J 1.54g software enabled visualization of the surface (surface plot) and cross-sectional (plot profile) structures (Figure 10). The plots depicted intensities as gray values on the *y*-axis, with more packed arrangements in semi-crystalline PLA compared to amorphous PLA. The gaps are more common in Proteinase K-treated PLA films.

## 4. Discussion

Biopolymers, such as PLA, are increasingly popular worldwide due to their excellent properties, including biocompatibility, mechanical strength, and biodegradability [6]. However, PLA is not biodegradable under normal ambient conditions. PLA polymers are largely resistant to attack by soil microorganisms, and PLA degradation upon disposal in the environment is challenging [24]. While some microorganisms are capable of degrading PLA under optimized conditions, several studies have illustrated the PLA degradation by Proteinase K [9,25,26] as an eco-friendly biocatalyst [11,12]. In this study, we further investigated the enzymatic degradation of both amorphous and semi-crystalline PLA using Proteinase K at 37 °C.

Quantifying the weight loss is the simplest and most direct method to quantify the degradation of the polymer, and weight loss is directly related to the surface area of the polymer pieces [27]. Our results showed that weight loss occurred in both amorphous and semi-crystalline PLA, with no statistically significant differences after 4 h for amorphous PLA and 48 h (2 days) for semi-crystalline PLA. The findings align with the previous study, which illustrated similar trends in weight loss after 1 day and 2 days of enzymatic treatment, using enzyme concentrations of 0.1 mg/mL and 0.5 mg/mL, respectively [26]. Amorphous PLA (characterized by low crystallinity) degraded significantly faster than semi-crystalline PLA, aligning with findings from earlier studies [9,25]. It has also been reported that the materials with low crystallinity, including biodegradable polymer blends, undergo faster degradation [15,25]. The degradation behavior of the biodegradable polymer blends depends on the proportion of PLA mixed in the blend, and the enzymatic degradation of the blends was found to be accelerated with less than 40% of PLLA in the blend [15]. Furthermore, Proteinase K has been shown to preferentially degrade the L-isomer of PLA over D-PLA [9].

In our study, a notable weight loss in amorphous PLA was observed just after 4 h, while semi-crystalline PLA showed changes only after 2 days. The higher weight loss in amorphous PLA might be because water in amorphous PLA can easily diffuse into the polymer matrix and react with the ester linkages, resulting in erosion and degradation. This initial stage degradation might be due to the initial active action of Proteinase K, causing exterior erosion of the PLA [1]. In contrast, the more ordered structure of semi-crystalline polymers slows down water permeation, leading to slower hydrolysis and delayed degradation [28]. However, while weight loss provides a useful preliminary measure of degradation, it alone does not fully capture the complexity of the biodegradation process. Other complementary analytical techniques are required to gain a comprehensive understanding of the changes occurring during PLA degradation.

A significant drop in the pH of the buffer solution was observed during enzymatic treatment from pH 8.5 to 3.97 for amorphous and 4.95 for semi-crystalline PLA. This acidification is likely due to the release of lactic acid and/or the formation of acidic oligomers as byproducts of PLA degradation [29]. A clear link was observed between the final pH of the enzyme solution and the extent of PLA film weight loss, supporting pH-dependent hydrolysis of PLA [22,26]. Amorphous PLA degrades faster because its polymer chains are more loosely and irregularly packed, allowing water to penetrate easily, and enhancing the enzymatic reaction [28,29,30,31]. Furthermore, hydrolysis of ester bonds during degradation leads to an increased concentration of carboxylic acid groups. These acidic moieties contribute to autocatalytic hydrolysis, further promoting the breakdown of PLA [28].

The concentrations of lactic acid were quantitatively analyzed using HPLC, and results showed a time-dependent increase in lactic acid production during enzymatic treatment. However, the concentrations of lactic acid were not significantly different after 8 h of incubation for amorphous PLA and 3 days for semi-crystalline PLA. The increase in lactic acid production increases the acidity of the medium, which may denature the enzyme, possibly resulting in non-significant weight loss after a certain time [26]. The results suggest that the degradation of PLA primarily occurs in a few hours of treatment, with amorphous PLA degrading more rapidly than semi-crystalline PLA. Furthermore, the rate of LA production decreased over time, possibly due to product accumulation or enzyme inactivation.

Our study also revealed that replacing the enzyme solution three additional times after the initial incubation of 4 days resulted in greater weight loss of PLA films compared to using a single enzyme exposure. This approach did not significantly change pH and showed only a slight decrease in LA production. This enhanced degradation was likely due to the removal of the denatured enzyme that had become denatured by the acidic environment and the introduction of the fresh and active enzyme. However, after a certain point, the pH and lactic acid production stabilized, becoming similar to those observed with a single enzyme exposure [26].

SEM analyses revealed significant surface changes on the PLA film following enzymatic treatment. Initially, the PLA surfaces had a clear and smooth appearance, but post-enzymatic treatment showed increased roughness, cracks, and small pores on the PLA surface, which are indicative of the enzymatic degradation. These surface features also represent an increase in surface area, and the formation of pits and cracks is associated with progressive degradation [24,27]. The Image J plot profile and surface profile analyses visualized the changes in their structural appearance by plotting two-dimensional and three-dimensional graphs. A more stacked or packed structure was observed in semi-crystalline samples and in samples subjected to repeated enzymatic treatments. This may result from the degradation of the PLA matrix, which leaves more space behind and leads to the formation of more densely packed and porous structures.

The change in the structure of PLA was further examined using XRD analysis. Since the intensity of diffraction peaks in XRD is related to crystallinity, this technique is commonly used to assess the crystallinity of PLA films. High-crystallinity materials typically exhibit relatively sharp, well-defined peaks [11] and are expected to decrease in peak intensity after enzymatic treatment for semi-crystalline PLA and a change from an ordered structure to an amorphous structure, and vice versa. In our study, sharp diffraction peaks were observed only in semi-crystalline PLA chromatograms, while amorphous PLA chromatograms displayed broader, less defined peaks. However, no significant changes in XRD patterns were observed before and after enzymatic treatment for either type of PLA, indicating that overall crystallinity remained unchanged in both PLA types.

PLA films (amorphous and semi-crystalline), after enzymatic treatment, were thermally analyzed using DSC and found no change in melt temperature (Tm) and glass transition temperature (Tg) among amorphous PLA films and semi-crystalline PLA film samples. Similar results were observed in [22]. The endothermic peak, the implied Tm of both amorphous and semi-crystalline PLA, was observed around 175–176 °C and refers to the high stereoregularity of the polymer chains in crystalline PLA [9]. When the trend in the crystallinity of (both amorphous and crystalline) PLA was analyzed, the trends were found to be almost constant and not significantly different. Diani and Gall [29] reported that the crystallinity percent was irregular for PLA32 and PLA118 samples; both samples peaked between 5 and 6 days, and a drop in crystallinity was observed at 14 days.

Limsukon et al. [32] also observed that as the amorphous regions of PLA polymer are degraded, the remaining polymer chains realign tightly to maintain a high degree of crystallinity [32]. Nguyen et al. reported that microbial degradation of Low-Density Polyethylene (LDPE) preferentially attacks the amorphous regions of the polymer. The crystalline regions of the LDPE were not degraded, and consequently, the overall percent crystallinity of the material increased [33]. Thus, the percentage of crystallinity of polymers may be a major limitation for the biodegradation of plastic materials such as PLA.

## 5. Conclusions

PLA is biodegradable under industrial composting systems, as well as at temperatures at or above its Tg (58 ± 5 °C), but it is highly resistant to biodegradation under mesophilic temperatures. The high production and current waste management practices for PLA may contribute to the global plastic pollution problem. Therefore, this study explored a method to degrade PLA under ambient conditions that will aid in the mitigation of PLA-based plastic pollution. The present study concluded that Proteinase K (2 mg/mL) has the potential to effectively degrade PLA films with both a low percentage of crystallinity (0–3%, referred to as amorphous PLA) and a high percentage of crystallinity (47–50%, referred to as semi-crystalline PLA) at 37 °C, a temperature significantly lower than required for industrial composting of PLA.

After 8 days of Proteinase K treatment, amorphous PLA exhibited greater degradation, with 15.2% weight loss and ~0.14 mMol/mL lactic acid monomer release, compared to semi-crystalline PLA, which exhibited 10.3% weight loss and ~0.07 mMol/mL lactic acid monomer release. This also resulted in a greater pH drop for amorphous PLA (pH 8.5 to 3.97). Repeated enzymatic treatment of PLA (four additions of fresh enzyme solution over 96 h) resulted in an increase in weight loss for both the amorphous (72.73%, 4.8 times) and semi-crystalline (39.75%, 4.2 times) PLA, indicating that repeatedly changing the enzyme solution enhanced the degradation of PLA.

Surface morphology analysis using SEM and Image J analyses of the SEM images (surface profiles and plot profiles) revealed substantial changes in amorphous PLA compared to semi-crystalline PLA. Thermal (DSC) and structural (XRD) analyses confirmed that the percent crystallinity of both the amorphous and semi-crystalline PLA did not change over the course of Proteinase K treatment. Together, these data provide evidence that enzymatic degradation of PLA polymers by Proteinase K preferentially targets the amorphous regions of the polymers. 

## Data Availability

The original contributions presented in the study are included in the article/Supplementary Material, further inquiries can be directed to the corresponding author.

## Supplementary Materials

The following supporting information can be downloaded at https://www.mdpi.com/article/10.3390/polym17223042/s1. Figure S1. SEM pictures of PLA films of 0 h, 1 h, 4 h, 8 h, 24 h, 96 h, 192 h, and 4 × 96 h (h: hour, 4 × 96 h: 4 × 4 days) Proteinase K treatment.
