# Supplementary material for: Enzymatic Degradation of PLA: Preferential Degradation of the Amorphous Fraction
Source: Polymers (Basel). 2025 Nov 17;17(22):3042. doi: 10.3390/polym17223042 (PMC12655971; doi:10.3390/polym17223042)
Supplement: Supplementary file 1 [file polymers-17-03042-s001.zip › polymers-3866071-supplementary.pdf]

Supplementary Material

Supplementary Figure

The SEM micrographs depict the surface morphology of the PLA films before and after Proteinase K treatment at 37 °C for different time periods.

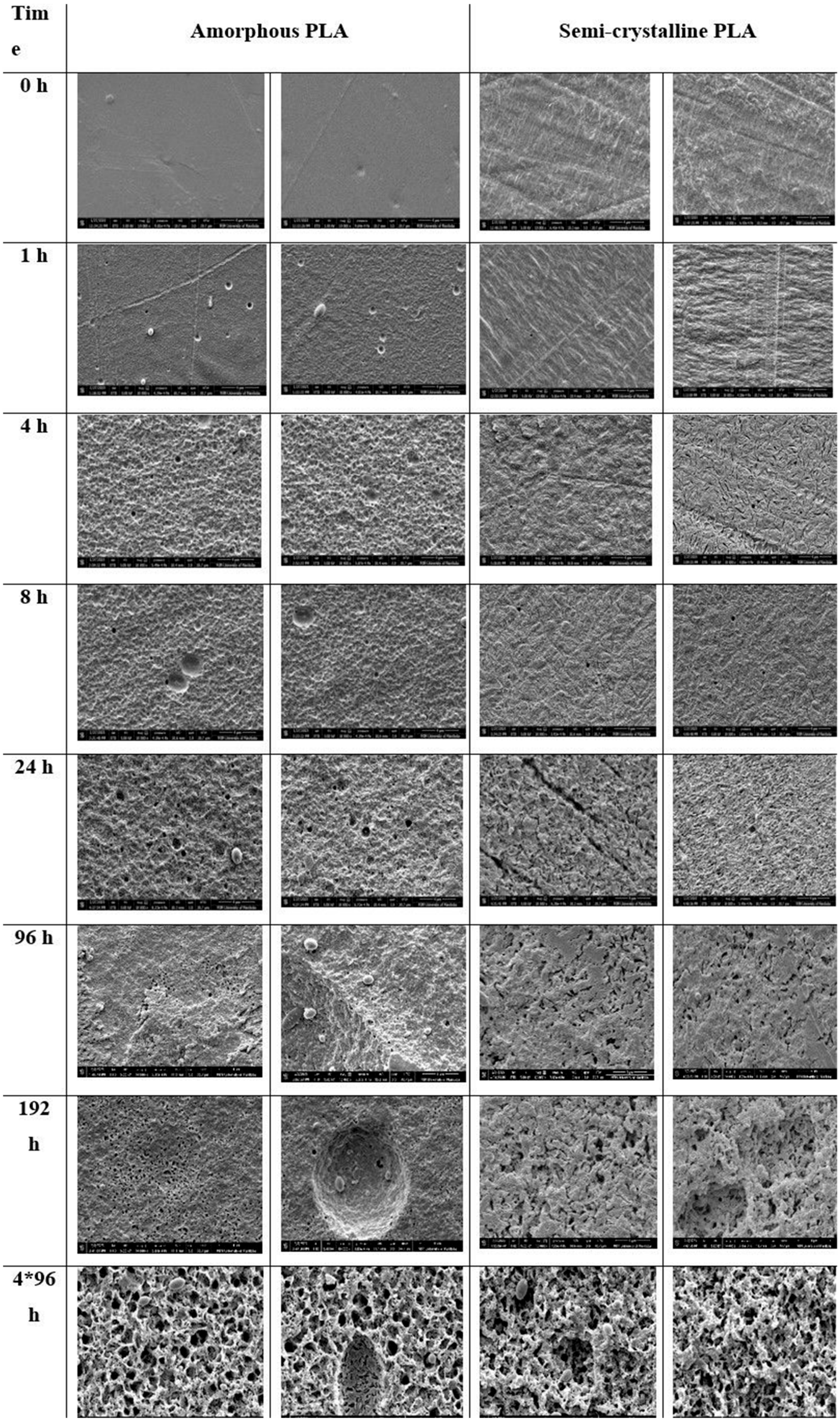

Figure S1. SEM pictures of PLA films of 0 h, 1 h, 4 h, 8 h, 24 h, 96 h, 192 h, and 4x96 h (h: hour, 4x96 h: 4x4 days) Proteinase K treatment
